# ESBL-Producing *Klebsiella pneumoniae* Bacteraemia with Renal Micro-Abscesses Treated with Cefepime–Enmetazobactam in a Neutropenic Allogeneic Transplant Recipient

**DOI:** 10.3390/pathogens15090976

**Published:** 2026-09-14

**Authors:** Carlo Tascini, Luca Montanari, Francesca Patriarca, Michela Bulfoni, Renato Fanin, Simone Giuliano, Jacopo Angelini, Paolo Gaibani

**Affiliations:** 1 Infectious Diseases Clinic, Azienda Sanitaria Universitaria Friuli Centrale (ASUFC), 33100 Udine, Italy; carlo.tascini@uniud.it (C.T.);; 2 Department of Medicine (DMED), University of Udine, 33100 Udine, Italy; 3 Haematological Clinic and Transplant Centre, Azienda Sanitaria Universitaria Friuli Centrale (ASUFC), 33100 Udine, Italy; 4 Department of Laboratory Medicine, Azienda Sanitaria Universitaria Friuli Centrale (ASUFC), 33100 Udine, Italy; 5 Clinical Pharmacology and Toxicology Institute, University Hospital Friuli Centrale, Azienda Sanitaria Universitaria Friuli Centrale (ASUFC), 33100 Udine, Italy; 6 Department of Diagnostic and Public Health, University of Verona, 37100 Verona, Italy; paolo.gaibani@univr.it; 7 Microbiology and Virology Unit, Department of Pathology, Azienda Ospedaliera Universitaria Integrata di Verona, 37100 Verona, Italy

**Keywords:** cefepime–enmetazobactam, ESBL, *Klebsiella pneumoniae* ST307, OXA-1, SHV-28, renal abscess, neutropenia, allogeneic stem cell transplantation, microbiome, therapeutic drug monitoring, time to positivity

## Abstract

Extended-spectrum β-lactamase (ESBL)-producing *Klebsiella pneumoniae* causes difficult-to-treat bloodstream infections in patients with haematological malignancies, particularly after allogeneic haematopoietic stem cell transplantation (allo-HSCT). Carbapenems remain reliable, but their anti-anaerobic activity may aggravate intestinal dysbiosis and increase selection pressure for carbapenem resistance. Cefepime–enmetazobactam is a novel fourth-generation cephalosporin/β-lactamase inhibitor combination active against many class A ESBL-producing Enterobacterales. We describe a profoundly immunocompromised patient with acute myeloid leukaemia after allo-HSCT, grade III steroid-refractory gastrointestinal graft-versus-host disease, and intestinal colonization by an ESBL-producing *K. pneumoniae* isolate resistant to ceftolozane–tazobactam. The patient developed persistent bacteraemia and right pyelonephritis with small renal abscess-like lesions. Meropenem was rapidly de-escalated to cefepime–enmetazobactam, with temporary adjunctive fosfomycin and subsequently tigecycline. Blood-culture time to positivity progressively lengthened from 1.18 h to 16 h before cultures became negative. Serial cefepime therapeutic drug monitoring permitted repeated assessment of exposure and neurological toxicity risk. Whole-genome sequencing identified *K. pneumoniae* ST307 carrying *bla*_CTX-M-15_, *bla*_TEM-1_, *bla*_SHV-28_, and *bla*_OXA-1_, together with multiple resistance determinants and a large conjugative plasmid. This case describes the use of cefepime–enmetazobactam as part of a targeted carbapenem-sparing strategy for invasive ESBL-producing *K. pneumoniae* infection in a highly immunocompromised allo-HSCT recipient.

## 1. Introduction

Bloodstream infections (BSIs) caused by extended-spectrum β-lactamase (ESBL)-producing Enterobacterales are a major complication in patients with haematological malignancies and are associated with high morbidity and mortality. In this population, mucosal injury, prolonged or profound neutropenia, disruption of epithelial barriers, vascular devices, broad-spectrum antibiotic exposure, and immunosuppression favour bacterial translocation and the development of invasive and deep-seated infections. The gastrointestinal tract represents the main reservoir for Enterobacterales, and intestinal colonization by multidrug-resistant organisms may precede subsequent bloodstream infection. After allogeneic haematopoietic stem cell transplantation (allo-HSCT), gastrointestinal graft-versus-host disease (GVHD) further disrupts the intestinal barrier and may amplify the risk of bacterial translocation. In this setting, antimicrobial selection must therefore consider not only in vitro susceptibility but also the presumed infection source, bacterial burden and inoculum, resistance mechanism, pharmacokinetic variability, toxicity, potential for resistance selection, and ecological effects on the intestinal microbiome [1,2]. Carbapenems remain a standard treatment for severe ESBL-producing Enterobacterales infections. However, prolonged exposure may increase selective pressure for carbapenem-resistant organisms and contribute to disruption of the intestinal microbiota. In allo-HSCT recipients, loss of microbial diversity and depletion of anaerobic commensals have been associated with adverse outcomes, including an increased risk and severity of GVHD. Therefore, when supported by antimicrobial susceptibility data, targeted carbapenem-sparing strategies may be considered to limit unnecessary broad-spectrum exposure [1,2,3,4,5,6]. This consideration is particularly relevant when an effective targeted alternative to carbapenems is available on the basis of antimicrobial susceptibility testing and the underlying resistance mechanism.

Cefepime–enmetazobactam combines a fourth-generation cephalosporin with a penicillanic-acid sulfone β-lactamase inhibitor. Enmetazobactam has potent activity against several clinically important class A ESBLs, including CTX-M, TEM, and SHV enzymes, thereby restoring the activity of cefepime against many ESBL-producing Enterobacterales. Preclinical pharmacokinetic/pharmacodynamic studies have provided evidence supporting the combination against ESBL-producing *Klebsiella pneumoniae*, including in neutropenic infection models [7,8]. In the phase III ALLIUM trial, cefepime–enmetazobactam achieved a higher composite clinical and microbiological success rate than piperacillin–tazobactam in patients with complicated urinary tract infection (cUTI) and acute pyelonephritis, supporting its role as a potential carbapenem-sparing option for selected ESBL-producing infections [9]. At the same time, recent clinical data have further supported its activity against ESBL-producing Enterobacterales, although resistance may occur in isolates carrying additional β-lactamase mechanisms, emphasizing the importance of microbiological characterization and susceptibility testing [7,8]. Clinical evidence supporting cefepime–enmetazobactam in highly complex infections remains limited, particularly in allo-HSCT recipients with persistent bacteraemia and deep-seated infection. In such patients, altered pharmacokinetics may complicate antimicrobial exposure, and therapeutic drug monitoring may therefore assist treatment optimization. *K. pneumoniae* ST307 is a globally disseminated high-risk clone frequently associated with multidrug resistance and carriage of ESBL determinants, particularly blaCTX-M-15. Genomic characterization may therefore provide additional information on the resistance background of invasive isolates and support interpretation of antimicrobial susceptibility results. Against this background, we describe the use of cefepime–enmetazobactam in an allo-HSCT recipient with persistent ESBL-producing *K. pneumoniae* ST307 bacteraemia and renal micro-abscesses, integrating microbiological monitoring, cefepime TDM, and whole-genome sequencing.

## 2. Results

### 2.1. Case Presentation

A 60-year-old woman with adverse-risk acute myeloid leukaemia (AML) was diagnosed in August 2025. Molecular characterization showed myelodysplasia-related gene mutations, mutated CEBPA, RUNX1, and FLT3-ITD, with a normal karyotype and WT1 overexpression. The leukaemia was refractory to standard induction therapy with cytarabine and daunorubicin (7 + 3) plus midostaurin. Salvage treatment with azacitidine, venetoclax, and gilteritinib was subsequently administered. Given the high-risk disease characteristics and the inadequate response to induction therapy, the patient proceeded to allogeneic haematopoietic stem cell transplantation.

On 18 February 2026, the patient underwent allogeneic peripheral-blood haematopoietic stem cell transplantation from a 10/10 HLA-matched unrelated donor. The post-transplant course was complicated by grade III steroid-refractory acute graft-versus-host disease (GVHD) involving the skin and both the upper and lower gastrointestinal tract. The combination of severe gastrointestinal mucosal injury, profound immunosuppression, prolonged hospitalization, neutropenia, and anticipated repeated exposure to broad-spectrum antimicrobial agents placed the patient at particularly high risk of invasive bacterial infection. At the same time, the extensive gastrointestinal involvement represented a clinically relevant risk factor for intestinal barrier disruption and bacterial translocation, while repeated antimicrobial exposure increased the potential for further microbiome disruption and selection of multidrug-resistant organisms.

Cultures obtained from a rectal surveillance swab documented gastrointestinal colonization with ESBL-producing *K. pneumoniae*. Notably, the colonizing isolate was resistant to ceftolozane–tazobactam, indicating a multidrug-resistant phenotype and limiting the reliability of this agent as a potential carbapenem-sparing option. On 3 April 2026, urine culture yielded ESBL-producing *K. pneumoniae.* On 13 April 2026, the patient deteriorated rapidly, developing haemodynamic instability with hypotension, and blood cultures yielded ESBL-producing *K. pneumoniae*. In contrast to the surveillance isolate, the bloodstream isolate was reported to be susceptible to ceftolozane–tazobactam. Because of the severity of the clinical presentation and the need for immediately reliable ESBL coverage, meropenem was initiated empirically. After antimicrobial susceptibility results became available and following infectious-diseases consultation, meropenem was replaced on 14 April, after only 24 h of treatment, with cefepime–enmetazobactam as a targeted carbapenem-sparing regimen. This strategy was selected on the basis of the susceptibility profile of the bloodstream isolate and the need to provide effective anti-ESBL therapy while limiting unnecessary prolonged carbapenem exposure in a profoundly immunocompromised allo-HSCT recipient.

The initial bacteraemic burden appeared high. The shortest blood-culture time to positivity (TTP) recorded on 14 April was 1.18 h, consistent with a substantial circulating bacterial burden. Despite administration of active antimicrobial therapy, repeat blood cultures initially remained positive. Adjunctive fosfomycin (8 g loading dose followed by 12 g/24 h by continuous intravenous infusion) was therefore administered from 19 to 23 April 2026. In vitro fosfomycin susceptibility of the bloodstream *K. pneumoniae* isolate was not tested before or during treatment. Subsequently, the patient showed clinical improvement accompanied by a progressive decline in inflammatory biomarkers. At the onset of bacteraemia, C-reactive protein (CRP) was 205 mg/L, and procalcitonin (PCT) was 35.94 ng/mL; both progressively declined and normalized by early May. Importantly, serial blood cultures demonstrated a marked prolongation of TTP, increasing from 1.18 h on 14 April to 12 h on 19 April and 16 h on 26 April (Table 1).

Although a microbiological cure had not yet been achieved, this progressive delay in blood-culture positivity was interpreted as a finding compatible with a substantial reduction in the circulating bacterial burden. Persistent bacteraemia nevertheless prompted further investigation for an uncontrolled anatomical or intravascular source, particularly given the prolonged positivity despite an apparently active antimicrobial regimen.

Abdominal computed tomography (CT) demonstrated right-sided pyelonephritis with small hypodense renal lesions consistent with early renal abscess formation. The renal findings provided a plausible deep-seated source for the persistent bacteraemia and represented a potential anatomical explanation for the delayed microbiological clearance. Urological assessment found no drainable collection and no indication for surgical intervention or percutaneous drainage. Conservative management was therefore pursued with continued antimicrobial therapy and close clinical and radiological follow-up. During the course of treatment, *Enterococcus faecium* was additionally isolated from blood cultures. Fosfomycin was consequently discontinued, and tigecycline (100 mg loading dose followed by 50 mg every 12 h.) was administered for 10 days, always in association with cefepime–enmetazobactam. In parallel, all vascular-access devices were subsequently replaced, addressing a possible secondary intravascular reservoir and providing an additional source-control measure. The first negative blood cultures were obtained on 29 April.

The patient remained persistently neutropenic during most of the infectious episode and received ongoing granulocyte colony-stimulating factor support. Neutrophil recovery occurred approximately one week before completion of cefepime–enmetazobactam therapy.

Because management included cefepime–enmetazobactam, temporary adjunctive fosfomycin, tigecycline for *E. faecium*, replacement of vascular-access devices, and recovery of the host immune status, the favourable outcome cannot be attributed to a single intervention. Nevertheless, cefepime–enmetazobactam represented the principal targeted anti-ESBL backbone of therapy after the initial 24 h of meropenem and allowed avoidance of prolonged carbapenem exposure.

### 2.2. Therapeutic Drug Monitoring Results

Serial cefepime TDM was conducted throughout treatment to evaluate antimicrobial exposure in the context of potentially variable renal clearance and to support assessment of both efficacy and toxicity.

Given the recent introduction of cefepime/enmetazobactam into clinical practice, PK/PD evidence remains limited, and no specific guidelines, consensus statements, or position papers currently define validated PK/PD efficacy and safety targets specifically for cefepime/enmetazobactam. Accordingly, our TDM-guided therapy was based on cefepime concentrations interpreted according to EUCAST clinical breakpoints and the isolate-specific MIC, with the aim of maintaining unbound cefepime concentrations at least fourfold above the MIC throughout the entire dosing interval. This aggressive PK/PD target was selected to increase the probability of clinical and microbiological success while minimizing the risk of antimicrobial resistance emergence [10,11,12]. To enable a more accurate interpretation of measured total plasma drug concentrations, the estimated 80% unbound fraction of cefepime and changes in the patient’s renal function were also considered, as both protein binding and renal function are known to substantially influence β-lactam pharmacokinetics [12,13].

**Figure 1:**
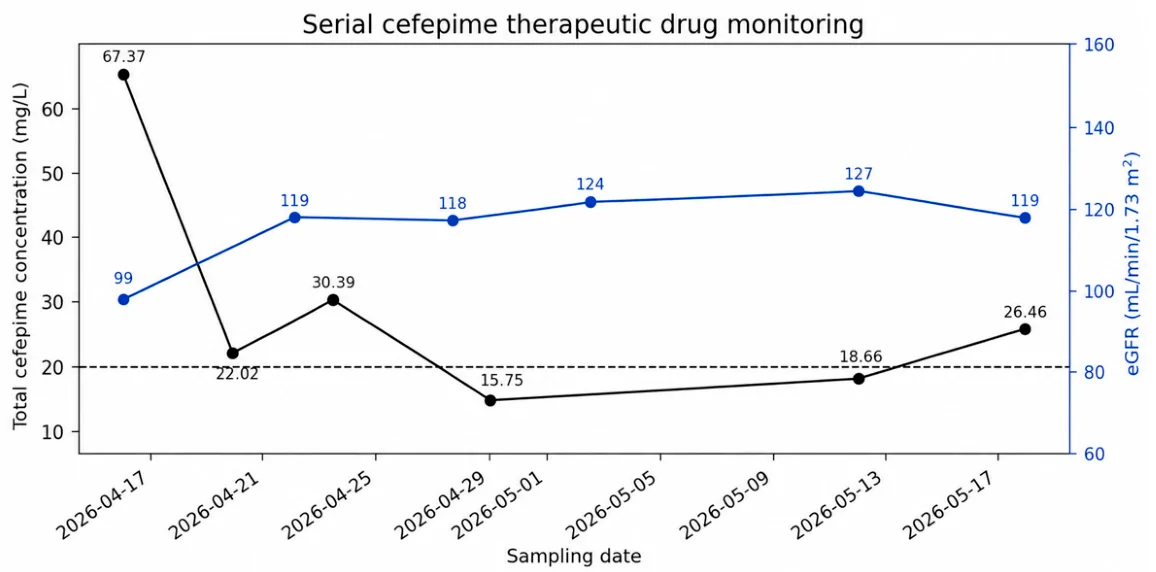
Serial pre-dosing total cefepime concentrations and estimated glomerular filtration rate (eGFR) during cefepime–enmetazobactam therapy. Black circles and line represent total cefepime concentrations, whereas blue circles and line represent eGFR values. The dashed line at 20 mg/L is shown only as a commonly discussed cautionary trough threshold for empiric treatments; not all samples were documented as true troughs, so direct application of trough-based toxicity cut-offs is inappropriate. The cefepime–enmetazobactam regimen was 1.25 g every 6 h over 4 h until 20 April and was subsequently adjusted to 1.25 g every 8 h over 4 h from 23 April onward on the basis of TDM.

Total pre-dosing cefepime concentrations were 67.37 mg/L on 16 April, 22.02 mg/L on 20 April, 30.39 mg/L on 23 April, 15.75 mg/L on 29 April, 18.66 mg/L on 12 May, and 26.46 mg/L on 18 May (Figure 1).

The lowest observed total concentration, 15.75 mg/L, corresponded to approximately 12.6 mg/L of unbound cefepime. Since the cefepime–enmetazobactam MIC of the bloodstream isolate was 0.25 mg/L, even the lowest measured cefepime concentration exceeded the MIC by approximately 50-fold and 4 × MIC by approximately 12.6-fold. Thus, all measured concentrations were compatible with attainment of the predefined PK/PD targets of 100% fT > MIC and 100% fT > 4 × MIC, provided that the lowest measured concentration approximated the minimum concentration during the relevant dosing interval [14,15,16,17,18,19,20].

In light of these considerations, together with the patient’s renal function and clinical course, we decided not to increase the cefepime/enmetazobactam dose, as a precautionary measure to avoid compromising the safety profile of the treatment.

The serial measurements also demonstrated marked intra-patient variability in cefepime exposure and supported repeated evaluation of dose, sampling time, renal clearance, target attainment, and potential neurotoxicity. No neurological syndrome attributable to cefepime was documented during the period covered by this report. Relative to the cefepime–enmetazobactam MIC of 0.25 mg/L, total concentration-to-MIC ratios ranged from 63 to 269. Even after applying a conservative free fraction of 80%, the lowest observed free concentration-to-MIC ratio was approximately 50, strongly supporting PK/PD target attainment throughout treatment. At the same time, several measured concentrations were above conservative neurotoxicity warning thresholds, supporting the clinical value of safety-oriented TDM during prolonged high-dose cefepime therapy [14,15,16,17,18,19,20]. TDM therefore provided complementary information on the adequacy of antimicrobial exposure and the potential safety margin of treatment in a patient with dynamic renal function and a highly complex clinical course.

Follow-up abdominal CT performed on 12 May showed improved definition of the previously affected renal cortex and reduction in the suspected subcentimetre renal abscesses. The combined clinical, microbiological, and radiological evolution was therefore consistent with successful conservative management of a complicated renal source without the need for invasive drainage. The progressive prolongation of TTP, subsequent blood-culture sterilization, clinical improvement, and radiological regression of the renal lesions provided concordant evidence of treatment response. Cefepime–enmetazobactam was subsequently continued until 26 May 2026, for a total treatment duration of 42 days.

### 2.3. Phenotypic and Genomic Characteristics of K. pneumoniae Strains

The invasive isolate was classified as ESBL-producing and susceptible to cefepime–enmetazobactam (Table 2). It was resistant to piperacillin–tazobactam. The intestinal surveillance isolate was resistant to ceftolozane/tazobactam, whereas the bloodstream isolate was reported susceptible to ceftolozane/tazobactam. The phenotype was interpreted in conjunction with WGS. Particular attention was paid to β-lactamase genes capable of explaining the ESBL phenotype and β-lactam/β-lactamase inhibitor behaviour. The measured DNA concentration was 90.6 ng/µL. Quality control and assembly metrics included a Q30 rate of 88.1%, mean coverage of 62×, mean read length of 149 bp, estimated genome size of 5.53 Mb, N50 of 307 kb, and 67 contigs (Table 3).

**Figure 2:**
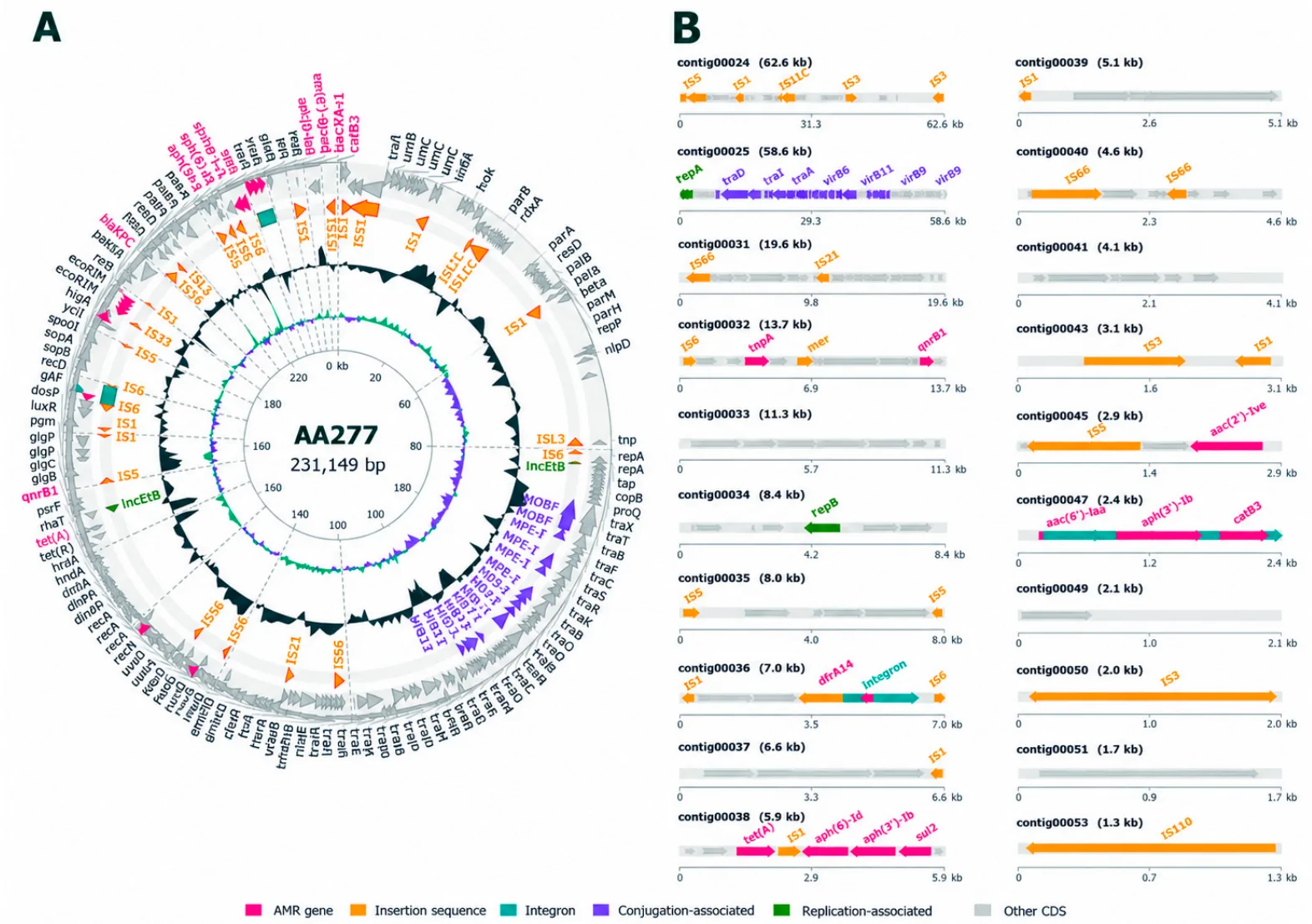
In silico reconstruction of the putative AA277 plasmid from Illumina short-read sequencing data. (**A**) Circular overview of the predicted 231,149-bp plasmid cluster generated using MOB-suite v3.1.9. (**B**) Linear representation of the 20 individual assembly contigs assigned to the same plasmid cluster, reconstructed from the GFF3 annotation. Colours indicate AMR genes (pink), insertion sequences (orange), integrons (turquoise), conjugation-associated genes (purple), replication-associated genes (green), and other coding sequences (grey). The circular map is a graphical representation of the in silico reconstruction and does not indicate that the plasmid was completely resolved or circularized. Accordingly, the physical linkage of all 11 AMR genes on a single plasmid molecule cannot be conclusively established from the available short-read data.

The isolate was assigned to ST307. Identified beta-lactamase genes were *bla*_CTX-M-15_, *bla*_TEM-1_, *bla*_SHV-28_, and *bla*_OXA-1_. Additional determinants were detected for aminoglycosides, fluoroquinolones, tetracyclines, trimethoprim, sulfonamides, chloramphenicol, and fosfomycin. A putative 231-kb IncFIB/IncFII conjugative plasmid carried multiple antimicrobial-resistance genes, indicating substantial potential for horizontal dissemination (Figure 2).

The potential significance of OXA-1 for piperacillin–tazobactam resistance, and of SHV-28 plus CTX-M-15 for expanded-spectrum cephalosporin resistance, was considered. Although aerobactin-associated genes were detected, the other investigated hyper-virulence-associated markers were not identified. Therefore, this finding alone was not considered sufficient to classify the ST307 isolate as hyper-virulent, particularly in the absence of phenotypic virulence assays.

## 3. Discussion

This case describes the use of cefepime–enmetazobactam in a particularly demanding clinical setting: an allo-HSCT recipient with steroid-refractory gastrointestinal GVHD, profound neutropenia, persistent ESBL-producing *K. pneumoniae* bacteraemia, and a renal parenchymal focus with early micro-abscess formation. Evidence for cefepime–enmetazobactam in such patients remains limited, and the available clinical data are largely derived from less complex populations. A literature search updated to 10 July 2026 identified a European case of ventilator-associated pneumonia caused by ESBL-producing *Escherichia coli* and a conference report describing ESBL-producing *E. coli* bloodstream infection in a patient with cirrhosis but no detailed peer-reviewed case involving a neutropenic patient with haematological malignancy or a recipient of allo-HSCT [21,22]. The present observation therefore expands the limited clinical experience with cefepime–enmetazobactam in severe ESBL-producing Enterobacterales infection while clearly not proving efficacy in comparable patients [23,24,25,26]. Rather, it provides a clinically detailed description of its use within a broader management strategy integrating antimicrobial susceptibility, source assessment, pharmacokinetic optimization, and microbiological monitoring.

Persistent bacteraemia in this patient did not necessarily indicate antimicrobial inactivity. Microbiological clearance may be delayed in the presence of a high initial inoculum, profound impairment of host defences, a poorly accessible tissue focus, or foreign material. These factors were simultaneously present or plausible in this patient, including profound neutropenia, gastrointestinal GVHD, renal parenchymal involvement with small abscess-like lesions, and indwelling vascular devices. The progressive prolongation of blood-culture time to positivity (TTP), together with clinical improvement and a decline in inflammatory biomarkers, suggested a progressive reduction in the circulating bacterial burden before definitive culture clearance. This observation is consistent with the concept that TTP may provide dynamic information during the course of bacteraemia, although it cannot replace conventional microbiological assessment. Importantly, continued blood-culture positivity appropriately prompted investigation for a renal or intravascular reservoir rather than immediate interpretation as failure of the antimicrobial regimen. The subsequent identification of pyelonephritis with suspected microabscesses and the replacement of vascular-access devices provided plausible explanations for the persistence of bacteraemia and reinforced the importance of source assessment in immunocompromised patients.

Whole-genome sequencing identified *K. pneumoniae* ST307 carrying *bla*_CTX-M-15_, *bla*_OXA-1_, *bla*_TEM-1_, *bla*_SHV-28_, fluoroquinolone resistance determinants, aminoglycoside-modifying enzymes, fosA, and a large predicted conjugative plasmid. The ST307 is a globally disseminated high-risk lineage that has been associated with multidrug resistance and invasive infections and may initially remain carbapenem-susceptible while retaining the capacity to acquire additional resistance determinants, including carbapenemases [27,28,29,30]. The genomic findings also have implications beyond the individual treatment episode. The presence of a high-risk clone, multiple resistance determinants, and a large predicted conjugative plasmid supports continued infection-control vigilance and surveillance for possible transmission and further resistance evolution, particularly in a high-risk transplant environment. A limitation of this study is that the putative plasmid was reconstructed exclusively from Illumina short-read data; therefore, long-read sequencing, such as Oxford Nanopore sequencing, and conjugation assays would be required to confirm its complete structure, the physical co-localization of the 11 AMR genes, and its functional transferability.

The piperacillin–tazobactam-resistant but cefepime–enmetazobactam-susceptible phenotype observed in the bloodstream *K. pneumoniae* isolate is biologically plausible. Co-production of OXA-1 with CTX-M-15, TEM-1, and SHV-28 can contribute to reduced susceptibility or resistance to piperacillin–tazobactam, particularly in isolates with a high β-lactamase burden and under high-inoculum conditions [31,32,33]. Enmetazobactam does not primarily target OXA-1; however, it provides potent inhibition of the dominant class A ESBL enzymes, particularly CTX-M enzymes, thereby protecting cefepime from hydrolysis. Cefepime also provides greater intrinsic stability against several ESBL-mediated hydrolytic mechanisms than piperacillin. The observed phenotype therefore illustrates an important principle in the management of multidrug-resistant Enterobacterales: β-lactam/β-lactamase inhibitor combinations cannot be considered interchangeable on the basis of their general class activity. The resistance mechanisms present in the individual isolate, together with the susceptibility result for the specific combination being considered, remain essential for clinical decision-making. This is particularly relevant for high-risk *K. pneumoniae* lineages carrying multiple β-lactamases, in which the coexistence of resistance determinants may generate phenotypes that cannot be reliably inferred from a single β-lactamase gene.

The discordance between the ceftolozane–tazobactam-resistant rectal surveillance isolate and the ceftolozane–tazobactam-susceptible bloodstream isolate is also noteworthy. Several mechanisms could account for this finding, including intestinal strain heterogeneity, differences in plasmid content, variation in β-lactamase expression, porin alterations, efflux mechanisms, or methodological differences between susceptibility determinations. In a patient with prolonged hospitalization and substantial antimicrobial exposure, colonization by more than one closely related or unrelated *K. pneumoniae* strain is biologically plausible. Surveillance cultures are useful for anticipating resistance and identifying patients at increased risk of infection by multidrug-resistant organisms, but they cannot replace antimicrobial susceptibility testing of the invasive isolate. Importantly, paired sequencing of the colonizing and bloodstream isolates was not available in the present case. This limitation prevents definitive determination of whether the two isolates represented the same strain with phenotypic differences, distinct members of the intestinal *K. pneumoniae* population, or sequential colonization and infection events. Paired genomic characterization would therefore have provided additional insight into the relationship between intestinal colonization and bloodstream infection.

The renal source provides a clinical link to the evidence generated by the ALLIUM trial, although the present patient was substantially more complex because of profound immunosuppression, persistent bacteraemia, and suspected renal micro-abscesses [9]. Initial meropenem therapy was used during acute clinical deterioration, followed by cefepime–enmetazobactam once pathogen identification and susceptibility data became available. The subsequent clinical course should nevertheless be interpreted cautiously. Fosfomycin was used temporarily as adjunctive therapy, tigecycline was administered for concomitant *E. faecium* bacteraemia, and vascular-access devices were replaced. Accordingly, the favourable outcome cannot be attributed to cefepime–enmetazobactam alone.

Broad-spectrum antibiotic exposure may affect intestinal microbial diversity in allo-HSCT recipients [3,4,5,6]. However, no microbiome assessment was performed in the present patient, and no ecological advantage of cefepime–enmetazobactam over prolonged carbapenem therapy can therefore be inferred from this case.

Treatment of bacteraemic pyelonephritis complicated by renal microabscesses requires adequate antimicrobial exposure at the site of infection and not only high urinary concentrations. Cefepime has low protein binding, distributes predominantly within extracellular fluid, and is largely eliminated by the kidneys, supporting its delivery to well-perfused renal tissue. Direct cefepime or enmetazobactam concentrations within the renal lesions were not available in this case, however, and antimicrobial penetration into small abscess cavities may be heterogeneous, particularly in the presence of necrosis, inflammation, or encapsulation. For small lesions not amenable to drainage, prolonged optimized antimicrobial exposure, repeated blood cultures, serial imaging, and continuous reassessment of source control are therefore central to management. In the present patient, urological evaluation did not identify a drainable collection, while follow-up CT on 12 May demonstrated improvement of the renal abnormalities and reduction of the suspected subcentimetre abscesses. The radiological response, together with microbiological clearance and clinical improvement, supported the decision to continue conservative management.

Serial TTP provided useful complementary information during the episode of persistent bacteraemia but should not be interpreted as a stand-alone quantitative measure of treatment response. TTP is influenced by several pre-analytical and analytical variables, including blood volume, sampling site, bottle type, transport and incubation conditions, and prior antimicrobial exposure [34,35]. The initial TTP of 1.18 h was unusually short but was confirmed against the original microbiology laboratory record. In the context of profound immunosuppression and persistent bacteraemia, this finding was considered biologically plausible and compatible with a high circulating bacterial burden. Nevertheless, the subsequent increase to 12 h on 19 April and 16 h on 26 April occurred in parallel with clinical and inflammatory improvement and was compatible with a progressive decline in circulating bacterial burden.

However, because pre-analytical variables were not prospectively standardized, this trend should be interpreted as complementary semi-quantitative information rather than as robust quantitative evidence of treatment response. Importantly, the delayed TTP did not justify premature classification as microbiological cure because cultures remained positive. Instead, it provided additional information supporting continued therapy while simultaneously reinforcing the need to investigate persistent anatomical or intravascular sources.

Therapeutic drug monitoring was particularly relevant because cefepime exposure must balance aggressive PK/PD targets against the risk of neurotoxicity. Cefepime-associated encephalopathy, myoclonus, seizures, and non-convulsive status epileptics are most strongly associated with excessive exposure and impaired renal clearance, although neurotoxicity may occur even when conventional renal function estimates appear acceptable [15,16,17,18,19,20]. Creatinine-based estimates may be particularly unreliable in patients with haematological malignancies, low muscle mass, rapidly changing renal function, or critical illness. In the present case, serial TDM demonstrated marked intra-patient variability in cefepime concentrations. The initial concentration of 67.37 mg/L was concerning and supported close reassessment of dose and exposure. Subsequent concentrations ranged from 15.75 to 30.39 mg/L. With an MIC of 0.25 mg/L and an estimated unbound fraction of 80%, even the lowest measured concentration corresponded to approximately 50 × MIC and approximately 12.6 × 4 × MIC, supporting sustained attainment of the desired PK/PD targets despite dose adjustment. At the same time, several concentrations were above conservative neurotoxicity warning thresholds. Thus, TDM provided information not only supporting pharmacodynamic adequacy but also identifying a potential safety concern that justified continued neurological surveillance and individualized dosing.

The principal limitations of this report should be acknowledged. First, paired WGS of the rectal surveillance isolate was not available, preventing definitive assessment of the genetic relationship between intestinal colonization and bloodstream infection. Second, blood-culture collection and processing may introduce variability in TTP measurements, and the exceptionally short initial TTP warrants verification. Third, only total plasma cefepime concentrations were measured, whereas enmetazobactam plasma concentrations and cefepime/enmetazobactam concentrations in the renal parenchyma or suspected abscesses were not assessed. Consequently, neither the unbound plasma concentrations of both components of the β-lactam/β-lactamase inhibitor combination nor their tissue exposure could be directly evaluated. Fourth, the use of combination and adjunctive therapies, vascular-line replacement, and recovery of host defences preclude attribution of microbiological clearance and radiological improvement to a single antimicrobial intervention. Finally, the experience of a single patient cannot establish the efficacy, optimal duration, or safety of cefepime–enmetazobactam in allo-HSCT recipients.

Despite these limitations, this case provides a detailed description of cefepime–enmetazobactam use in a highly complex allo-HSCT recipient with invasive ESBL-producing *K. pneumoniae* infection. It also highlights the importance of integrating isolate-specific susceptibility testing, anatomical source assessment, microbiological monitoring, and individualized TDM in the management of persistent bacteraemia. Further clinical studies are required to define the efficacy, optimal exposure, safety, and tissue penetration of cefepime–enmetazobactam in haematological and transplant populations.

## 4. Conclusions

Cefepime–enmetazobactam was used as the targeted anti-ESBL backbone for persistent *K. pneumoniae* ST307 bacteraemia associated with pyelonephritis and early renal micro-abscesses in a profoundly neutropenic allo-HSCT recipient. In this particularly complex clinical setting, the case illustrates a management strategy based on rapid initiation of reliably active empirical therapy, followed by targeted treatment once pathogen identification and susceptibility data are available. The clinical course also highlights the importance of integrating conventional susceptibility testing with whole-genome sequencing to characterize the resistance background and to better understand the potential implications of high-risk clones and coexisting β-lactamase determinants.

Repeated assessment of the anatomical source and source-control options was essential in the setting of persistent bacteraemia and renal parenchymal involvement. Serial blood-culture TTP provided an additional dynamic marker of the evolution of bacteraemia, with progressive prolongation preceding definitive microbiological clearance; however, TTP should be interpreted as a complementary parameter rather than a surrogate for microbiological cure. Serial cefepime therapeutic drug monitoring was also useful in assessing PK/PD target attainment against the potential risk of cefepime-associated neurotoxicity, particularly in a patient with potentially fluctuating renal clearance and prolonged high-dose exposure.

Because the favourable outcome occurred in the context of adjunctive antimicrobial therapy, vascular-line replacement, and repeated source assessment, it cannot be attributed exclusively to cefepime–enmetazobactam. This case therefore provides a descriptive clinical experience rather than evidence of efficacy. Further studies are needed to define the efficacy, optimal exposure targets, safety profile, tissue penetration, and potential role of cefepime–enmetazobactam in haematological malignancy and transplant populations.

## 5. Materials and Methods

### 5.1. The Clinical Data Collection

Clinical information was reconstructed from the electronic medical record, including underlying haematological disease, transplant characteristics, immunosuppressive treatment, antimicrobial exposure, laboratory trends, radiological findings, microbiological results, vascular-access management, and clinical outcome. Persistent bacteraemia was defined as repeated recovery of the same species from blood cultures despite administration of at least one agent considered active in vitro.

Blood-culture TTP was defined as the interval between incubation of the blood-culture bottle in the automated system and detection of a positive signal. For the present analysis, the shortest available TTP associated with each clinically relevant sampling date was used. Serial values were interpreted as a semiquantitative marker of viable bloodstream bacterial burden while recognizing that TTP is influenced by blood volume, bottle type, previous antimicrobial exposure, sampling site, transportation delay, and instrument-related factors. The prespecified clinical timeline included TTP values of 1.18 h on 14 April, 12 h on 19 April, and 16 h on 26 April, followed by negative cultures on 29 April 2026.

### 5.2. Microbiological Characterization

Rectal surveillance and blood-culture isolates were identified by routine clinical microbiology methods. Briefly, microbial identification was carried out from overnight subcultures using matrix-assisted laser desorption ionization–time of flight mass spectrometry (MALDI-TOF MS, Bruker Daltonik GmbH, Bremen, Germany), antimicrobial susceptibility was performed on overnight subcultures using the automated VITEK^®^ 2 system with the GN card (BioMérieux, Lyon, France), and results were interpreted following the European Committee on Antimicrobial Susceptibility Testing (EUCAST). Susceptibility to cefepime and cefepime/enmetazobactam was assessed by MIC TestStrip (Liofilchem, Roseto Degli Abruzzi, Teramo, Italy) [36], and results were interpreted following the EUCAST clinical breakpoints (EUCAST v 16.0, 2026). Carbapenemase production was assessed using the rapid chromatographic assay NG Test^®^ CARBA 5 (NG Biotech, Guipry, France).

### 5.3. Whole-Genome Sequencing and Resistome Analysis

WGS was performed on the ESBL-producing bloodstream *K. pneumoniae* isolate as previously described [37]. Briefly, DNA was extracted using the QIAsymphony complex protocol. Libraries were prepared with the FX DNA Library Prep Kit (Qiagen, Hilden, Germany) and Nextera XT index reagents, and sequencing was performed on an Illumina MiSeq platform (Illumina, San Diego, CA, USA) using MiSeq Reagent Kit v.3 with 2 × 300 paired-end reads. Prior to genome assembly, a filtering step was conducted to remove any contaminant human reads. The read quality evaluation was performed with FastQC 0.11.9 (https://www.bioinformatics.babraham.ac.uk/projects/fastqc/, accessed on 1 January 2023) and aggregated MultiQC reporting before and after filtering. A minimum Q score of 30 was set to filter Illumina reads. Bioinformatic analysis used OmicsBox 3.4.5, FastQC 0.12.1, MLST 2.0, ResFinder 4.7.2, PlasmidFinder 2.0, MobileElementFinder, Kraken2 RefSeq 2024-11 WGS, and PubMLST. The analysis included species identification, multilocus sequence typing, resistance-gene detection, plasmid replicon and mobility assessment, mobile genetic elements, and selected virulence-associated loci.

### 5.4. Therapeutic Drug Monitoring (TDM) Analysis

Cefepime–enmetazobactam was administered as the targeted therapy, in extended infusion over 4 h, after initial meropenem treatment. Dose and infusion frequency were adjusted by the treating team according to renal function, clinical condition, and local practice. Serial TDM included measurement of total plasma cefepime concentrations only, using an IVDR-certified HPLC-UV assay (Chromsystems, Gräfelfing, Bavaria, Germany). TDM interpretation integrated the antimicrobial objective of maintaining unbound cefepime concentrations above the MIC throughout the dosing interval with the safety objective of avoiding sustained excessive exposure. Since no clear continuous toxicity threshold is available for extended infusion of cefepime, we prudentially considered an excessive exposure in case of total cefepime trough concentrations >35 mg/L [20]. For severe infection in a profoundly immunocompromised host, 100% fT > MIC was considered the minimum PK/PD target, while 100% fT > 4 × MIC was used as an intensified target. Cefepime protein binding was conservatively estimated at approximately 20%. Neurological status and renal function were reviewed throughout treatment because cefepime accumulation, especially in renal dysfunction, is associated with encephalopathy, myoclonus, seizures, and non-convulsive status epilepticus [15,16,17,18,19,20].

Contrast-enhanced abdominal computed tomography was used to investigate persistent bacteraemia and identified right-sided pyelonephritis with small hypodense cortical lesions interpreted as early abscess-like foci. Urological consultation assessed whether drainage or surgery was indicated. Because the lesions were subcentimetre and not considered amenable to intervention, conservative management was chosen. Follow-up computed tomography on 12 May evaluated radiological response. The antimicrobial strategy was therefore required to provide adequate serum exposure for bacteraemia and sufficient delivery to infected renal parenchyma, while the clinical team repeatedly reassessed the possibility of an undrained focus.

### 5.5. Review of the Literature

A focused literature search was performed in PubMed and Google Scholar through 10 July 2026 using combinations of the following terms: (“cefepime enmetazobactam” OR “cefepime/enmetazobactam”) AND (“case report” OR “bacteraemia” OR “bloodstream infection” OR “neutropenia” OR “haematologic malignancy” OR “leukaemia” OR “stem cell transplantation”). Reference lists of relevant reviews and case reports were also screened. Human reports describing clinical use of cefepime–enmetazobactam were included, with particular attention to patients with haematological malignancies or allo-HSCT. Preclinical studies were considered separately and were not classified as human clinical evidence [7,8,38].

## Figures and Tables

**Table 1:** Clinical, microbiological, and therapeutic course of persistent *K.pneumoniae* bacteraemia.

Date	Blood Culture/Time to Positivity (TTP)	Antimicrobial Treatment/Major Intervention	Relevant Clinical/Radiological Findings
13 April 2026	ESBL-producing *K. pneumoniae* bacteraemia	Meropenem initiated empirically	Rapid clinical deterioration in a profoundly immunocompromised allo-HSCT recipient
14 April 2026	Positive for ESBL-producing *K. pneumoniae*; shortest TTP 1.18 h	1.18 h meropenem discontinued after 24 h; cefepime–enmetazobactam started as targeted carbapenem-sparing therapy	Very short TTP consistent with a high circulating bacterial burden
During persistent bacteraemia	—	Urological evaluation; conservative management selected because no drainable collection was identified	Abdominal CT demonstrated right-sided pyelonephritis with small hypodense cortical lesions consistent with early renal abscess-like foci
19 April 2026	Positive for ESBL-producing *K. pneumoniae*; TTP 12 h	Cefepime–enmetazobactam continued; adjunctive fosfomycin added during persistent bacteraemia	Clinical improvement with progressive decline in inflammatory biomarkers; TTP prolongation suggested decreasing circulating bacterial burden
26 April–23 April 2026	Positive for ESBL-producing *K. pneumoniae*; TTP 16 h Enterococcus faecium additionally isolated from blood culture	16 h fosfomycin discontinued; tigecycline administered for 10 days in association with cefepime–enmetazobactam; all vascular-access devices subsequently replaced	—
29 April 2026	First negative blood cultures	Not applicable; cefepime–enmetazobactam continued	Microbiological clearance achieved
12 May 2026	—	Cefepime–enmetazobactam continued	Follow-up abdominal CT showed improved definition of the affected renal cortex and reduction of the suspected subcentimetre renal abscesses
26 May 2026	—	Cefepime–enmetazobactam discontinued	—

**Abbreviations:** allo-HSCT, allogeneic haematopoietic stem cell transplantation; ESBL, extended-spectrum β-lactamase; TTP, time to positivity.

**Table 2:** Antimicrobial susceptibility profile of the *K. pneumoniae* isolate.

MIC (mg/L)	Interpretation	Antibiotic
≤4	S	Amikacin
>64	R	Amoxicillin/clavulanate
64	R	Ceftazidime
≤1	S	Ceftazidime/avibactam
≤0.5	S	Ceftolozane/tazobactam
>4	R	Ceftriaxone
>1	R	Ciprofloxacin
≤0.5	S	Ertapenem
>8	R	Gentamicin
≤0.125	S	Meropenem
32	R	Piperacillin/tazobactam
>8	R	Trimethoprim/sulfamethoxazole
0.5	S	Cefepime/enmetazobactam

Abbreviations: MIC, minimum inhibitory concentration; S, susceptible; R, resistant.

**Table 3:** Microbiological and genomic characteristics of the bloodstream isolate.

Finding	Domain
*Klebsiella pneumoniae*, MLST ST307	Species/clone
*bla*_CTX-M-15_, *bla*_TEM-1_, *bla*_SHV-28_, *bla*_OXA-1_	Key β-lactamases
*aac(6′)-Ib-cr5*, *aac(3)-IIe*, *aph(3″)-Ib*, *aph(6)-Id*, *qnrB1*, *gyrA S83I*, *parC S80I*, *tet(A)*, *dfrA14*, *sul2*, *catB3*, *fosA*	Additional AMR determinants
ESBL production; piperacillin–tazobactam resistance; cefepime–enmetazobactam susceptibility	Phenotype of clinical relevance
Approximately 231 kb; 11 AMR genes reported	Predicted conjugative plasmid
Enterobactin, aerobactin-associated genes, type 1 and type 3 fimbriae, capsule/O-antigen loci, and type VI secretion system	Virulence-associated features
Aerobactin receptor gene *iutA* detected; the complete aerobactin locus was not demonstrated; other major markers, including *rmpA*, *rmpA2*, *iroBCDN*, and *peg-344*, not detected. Hypervirulence was not phenotypically assessed	Hypervirulence-associated markers
Illumina MiSeq; 62× coverage; Q30 88.1%; 5.53 Mb; N50 307 kb; 67 contigs	Sequencing quality

## Data Availability

The clinical and genomic data supporting this report are contained within the article. Raw sequencing data are not publicly available because of institutional data-governance restrictions. Additional information may be available from the corresponding author upon reasonable request, subject to institutional approval.
